# 4-Methyl-3-nitro­pyridin-2-amine

**DOI:** 10.1107/S1600536809022582

**Published:** 2009-06-20

**Authors:** Misbahul Ain Khan, M. Nawaz Tahir, Abdul Qayyum Ather, Maryam Shaheen, Rauf Ahmad Khan

**Affiliations:** a Institute of Chemistry, University of the Punjab, Lahore, Pakistan; b University of Sargodha, Department of Physics, Sargodha, Pakistan; c Applied Chemistry Research Center, PCSIR Laboratories complex, Lahore 54600, Pakistan

## Abstract

In the title compound, C_6_H_7_N_3_O_2_, the dihedral angle between the nitro group and the pyridine ring is 15.5 (3)° and an intra­molecular N—H⋯O hydrogen bond occurs. In the crystal, inversion dimers linked by two N—H⋯N hydrogen bonds occur, resulting in *R*_2_^2^(8) rings. The packing is stabilized by aromatic π–π stacking [centroid–centroid distance = 3.5666 (15) Å] and a short N—O⋯π contact is seen.

## Related literature

For a related structure, see: Kvick & Noordik (1977 ▶). For graph-set notation, see: Bernstein *et al.* (1995 ▶).
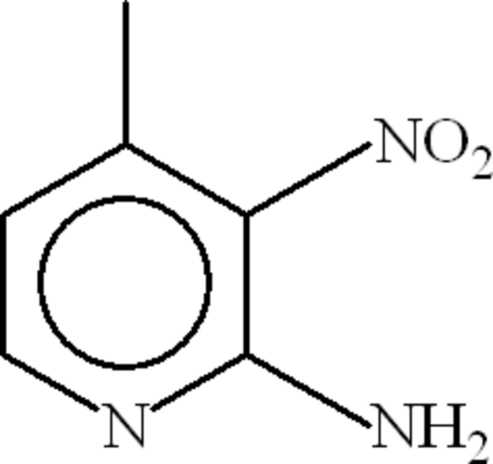


## Experimental

### 

#### Crystal data

C_6_H_7_N_3_O_2_
*M* *_r_* = 153.15
Monoclinic, 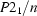

*a* = 7.3776 (6) Å
*b* = 12.8673 (11) Å
*c* = 7.3884 (6) Å
β = 104.364 (4)°
*V* = 679.45 (10) Å^3^
*Z* = 4
Mo *K*α radiation
μ = 0.12 mm^−1^
*T* = 296 K
0.25 × 0.10 × 0.08 mm

#### Data collection

Bruker Kappa APEXII CCD diffractometer
Absorption correction: multi-scan (*SADABS*; Bruker, 2005 ▶) *T*_min_ = 0.985, *T*_max_ = 0.992
7483 measured reflections
1677 independent reflections
759 reflections with *I* > 2σ(*I*)
*R*_int_ = 0.055

#### Refinement

*R*[*F* ^2^ > 2σ(*F* ^2^)] = 0.056
*wR*(*F* ^2^) = 0.173
*S* = 1.00
1677 reflections
107 parameters
H atoms treated by a mixture of independent and constrained refinement
Δρ_max_ = 0.39 e Å^−3^
Δρ_min_ = −0.32 e Å^−3^

### 

Data collection: *APEX2* (Bruker, 2007 ▶); cell refinement: *SAINT* (Bruker, 2007 ▶); data reduction: *SAINT*; program(s) used to solve structure: *SHELXS97* (Sheldrick, 2008 ▶); program(s) used to refine structure: *SHELXL97* (Sheldrick, 2008 ▶); molecular graphics: *ORTEP-3* (Farrugia, 1997 ▶) and *PLATON* (Spek, 2009 ▶); software used to prepare material for publication: *WinGX* (Farrugia, 1999 ▶) and *PLATON*.

## Supplementary Material

Crystal structure: contains datablocks global, I. DOI: 10.1107/S1600536809022582/hb5007sup1.cif

Structure factors: contains datablocks I. DOI: 10.1107/S1600536809022582/hb5007Isup2.hkl

Additional supplementary materials:  crystallographic information; 3D view; checkCIF report

## Figures and Tables

**Table 1:** Hydrogen-bond geometry (Å, °)

*D*—H⋯*A*	*D*—H	H⋯*A*	*D*⋯*A*	*D*—H⋯*A*
N2—H2*A*⋯N1^i^	0.88 (3)	2.17 (4)	3.045 (4)	174 (3)
N2—H2*B*⋯O1	0.85 (3)	2.01 (3)	2.612 (4)	127 (2)
N3—O2⋯*Cg*1^ii^	1.20 (1)	3.27 (1)	3.681 (12)	100 (1)

Symmetry codes: (i) 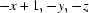; (ii) 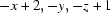. *Cg*1 is the centroid of the pyridine ring.

**Fig. 1.**
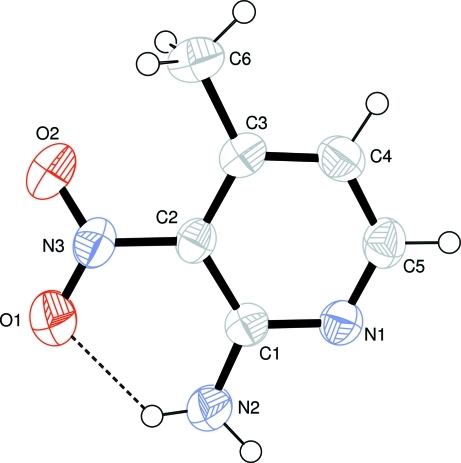
View of (I) with displacement ellipsoids drawn at the 50% probability level. H-atoms are shown by small spheres of arbitrary radii. Intermolecular H-bond is shown by dotted lines.

**Fig. 2.**
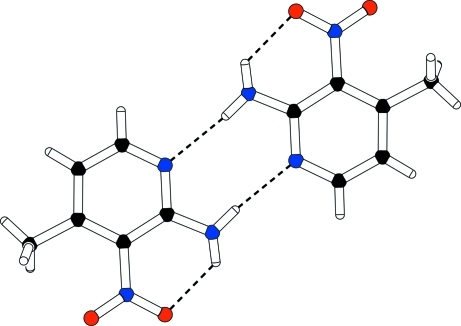
The partial packing of (I), which shows that molecules form dimers.

## supplementary crystallographic information

### Comment

Pyridines form a very important class of heterocyclic compounds. In it are included various vitamins, enzymes, pharmaceuticals, dyes, agrochemicals and other products. The title compound (I), (Fig. 1) is nitro substituted 2-Amino-4-methylpyridine.

The crystal structure of (II) 2-Amino-4-methylpyridine (Kvick & Noordik, 1977) has been reported. In (I), the pyridine ring A (C1—C5/N1) is planar with Rms deviation of 0.0135 Å. The amino N-atom and the methyl C-atom deviates from the plane of ring A by -0.0551 (37) Å and -0.044 (4) Å, respectively. The dihedral angle between ring A and nitro group B (O1/N3/O2) is 15.53 (27)°. The title compound consists of dimers due to inversion related intermolecular H-bonds of N–H···N type forming ring motifs *R*_2_^2^(8) (Bernstein *et al.*, 1995). The interamoleculr H-bond of N–H···O type completes *R*_1_^1^(6) ring motif (Fig. 2). The molecules are stabilized due to π–π-interactions with centroid to centroid distance of 3.5666 (15) Å [CgA···CgA^i^: symmetry code i = 2 - *x*, -*y*, -*z*] and N–O···π interactions (Table 1).

### Experimental

2-Amino-4-picoline (1.1 g, 0.01 mol) was dissolved in 10 ml of concentrated nitric and sulfuric acid (1:1) and cooled to 278 K. The mixture was left overnight and the resultant nitramino product was further treated with 5 ml of conc. sulfuric acid at room temperature for 3 h and poured over 250 g of crushed ice. The precipitates obtained were collected by filtration and subjected to steam distillation. The title compound was obtained as yellow needles of (I) on cooling the distillate to room temperature.

### Refinement

The coordinates of the H-atoms of the NH_2_ group were located in a difference map and refined. The other H-atoms were positioned geometrically (C—H = 0.93—0.96 Å) and refined as riding with U_iso_(H) = 1.2U_eq_(C, N).

### Crystal data

**Table:**

C_6_H_7_N_3_O_2_	*F*(000) = 320
*M**_r_* = 153.15	*D*_x_ = 1.497 Mg m^−^^3^
Monoclinic, *P*2_1_/*n*	Mo *K*α radiation, λ = 0.71073 Å
Hall symbol: -P 2yn	Cell parameters from 1677 reflections
*a* = 7.3776 (6) Å	θ = 3.2–28.3°
*b* = 12.8673 (11) Å	µ = 0.12 mm^−^^1^
*c* = 7.3884 (6) Å	*T* = 296 K
β = 104.364 (4)°	Needle, yellow
*V* = 679.45 (10) Å^3^	0.25 × 0.10 × 0.08 mm
*Z* = 4	

### Data collection

**Table:**

Bruker Kappa APEXII CCD diffractometer	1677 independent reflections
Radiation source: fine-focus sealed tube	759 reflections with *I* > 2σ(*I*)
graphite	*R*_int_ = 0.055
Detector resolution: 7.40 pixels mm^-1^	θ_max_ = 28.3°, θ_min_ = 3.2°
ω scans	*h* = −9→9
Absorption correction: multi-scan (*SADABS*; Bruker, 2005)	*k* = −17→17
*T*_min_ = 0.985, *T*_max_ = 0.992	*l* = −9→9
7483 measured reflections	

### Refinement

**Table:**

Refinement on *F*^2^	Primary atom site location: structure-invariant direct methods
Least-squares matrix: full	Secondary atom site location: difference Fourier map
*R*[*F*^2^ > 2σ(*F*^2^)] = 0.056	Hydrogen site location: inferred from neighbouring sites
*wR*(*F*^2^) = 0.173	H atoms treated by a mixture of independent and constrained refinement
*S* = 1.00	*w* = 1/[σ^2^(*F*_o_^2^) + (0.0745*P*)^2^ + 0.0769*P*] where *P* = (*F*_o_^2^ + 2*F*_c_^2^)/3
1677 reflections	(Δ/σ)_max_ < 0.001
107 parameters	Δρ_max_ = 0.39 e Å^−^^3^
0 restraints	Δρ_min_ = −0.31 e Å^−^^3^

### Special details

**Table:**

Geometry. Bond distances, angles *etc*. have been calculated using the rounded fractional coordinates. All su's are estimated from the variances of the (full) variance-covariance matrix. The cell e.s.d.'s are taken into account in the estimation of distances, angles and torsion angles
Refinement. Refinement of *F*^2^ against ALL reflections. The weighted *R*-factor *wR* and goodness of fit *S* are based on *F*^2^, conventional *R*-factors *R* are based on *F*, with *F* set to zero for negative *F*^2^. The threshold expression of *F*^2^ > σ(*F*^2^) is used only for calculating *R*-factors(gt) *etc*. and is not relevant to the choice of reflections for refinement. *R*-factors based on *F*^2^ are statistically about twice as large as those based on *F*, and *R*- factors based on ALL data will be even larger.

### Fractional atomic coordinates and isotropic or equivalent isotropic displacement parameters (Å^2^)

**Table:**

	*x*	*y*	*z*	*U*_iso_*/*U*_eq_	
O1	0.9915 (3)	−0.20091 (18)	0.2978 (3)	0.0860 (10)	
O2	1.2249 (3)	−0.11801 (19)	0.4483 (4)	0.0904 (10)	
N1	0.7197 (3)	0.06783 (18)	0.0831 (3)	0.0426 (8)	
N2	0.6845 (4)	−0.1039 (2)	0.1310 (3)	0.0529 (9)	
N3	1.0759 (3)	−0.11908 (19)	0.3358 (3)	0.0475 (9)	
C1	0.8004 (4)	−0.0221 (2)	0.1552 (3)	0.0386 (8)	
C2	0.9935 (3)	−0.0238 (2)	0.2507 (3)	0.0378 (9)	
C3	1.1041 (3)	0.0656 (2)	0.2608 (3)	0.0405 (9)	
C4	1.0135 (4)	0.1542 (2)	0.1803 (4)	0.0480 (10)	
C5	0.8246 (4)	0.1511 (2)	0.0979 (4)	0.0472 (10)	
C6	1.3108 (4)	0.0719 (3)	0.3480 (4)	0.0555 (10)	
H2A	0.570 (5)	−0.089 (2)	0.066 (4)	0.0635*	
H2B	0.730 (4)	−0.164 (2)	0.156 (4)	0.0635*	
H4	1.07986	0.21582	0.18170	0.0576*	
H5	0.76702	0.21275	0.04899	0.0566*	
H6A	1.35738	0.13785	0.31888	0.0666*	
H6B	1.37365	0.01708	0.29973	0.0666*	
H6C	1.33334	0.06475	0.48107	0.0666*	

### Atomic displacement parameters (Å^2^)

**Table:**

	*U*^11^	*U*^22^	*U*^33^	*U*^12^	*U*^13^	*U*^23^
O1	0.0667 (16)	0.0497 (16)	0.127 (2)	−0.0025 (12)	−0.0035 (14)	0.0240 (14)
O2	0.0609 (15)	0.0759 (19)	0.110 (2)	0.0105 (13)	−0.0247 (14)	0.0200 (14)
N1	0.0373 (12)	0.0424 (14)	0.0475 (13)	0.0041 (11)	0.0096 (10)	−0.0006 (11)
N2	0.0399 (13)	0.0506 (17)	0.0639 (16)	−0.0020 (13)	0.0048 (12)	0.0097 (14)
N3	0.0416 (14)	0.0502 (17)	0.0506 (14)	0.0091 (12)	0.0110 (12)	0.0076 (12)
C1	0.0355 (14)	0.0427 (16)	0.0394 (14)	0.0019 (13)	0.0126 (11)	−0.0016 (12)
C2	0.0356 (15)	0.0404 (16)	0.0378 (14)	0.0070 (12)	0.0101 (11)	0.0004 (12)
C3	0.0361 (14)	0.0500 (18)	0.0354 (14)	0.0038 (13)	0.0090 (11)	−0.0046 (12)
C4	0.0493 (18)	0.0399 (17)	0.0547 (17)	−0.0042 (14)	0.0126 (14)	−0.0032 (14)
C5	0.0495 (18)	0.0421 (17)	0.0492 (16)	0.0105 (14)	0.0110 (13)	−0.0001 (13)
C6	0.0391 (16)	0.066 (2)	0.0589 (18)	−0.0051 (14)	0.0077 (13)	−0.0058 (16)

### Geometric parameters (Å, °)

**Table:**

O1—N3	1.220 (3)	C2—C3	1.402 (4)
O2—N3	1.203 (3)	C3—C4	1.380 (4)
N1—C1	1.349 (3)	C3—C6	1.503 (4)
N1—C5	1.310 (4)	C4—C5	1.376 (4)
N2—C1	1.340 (4)	C4—H4	0.9300
N3—C2	1.442 (3)	C5—H5	0.9300
N2—H2B	0.85 (3)	C6—H6A	0.9600
N2—H2A	0.88 (3)	C6—H6B	0.9600
C1—C2	1.425 (4)	C6—H6C	0.9600
			
C1—N1—C5	118.4 (2)	C2—C3—C4	116.2 (2)
O1—N3—O2	119.7 (2)	C4—C3—C6	118.2 (3)
O1—N3—C2	119.9 (2)	C3—C4—C5	119.7 (2)
O2—N3—C2	120.4 (2)	N1—C5—C4	125.0 (3)
H2A—N2—H2B	126 (3)	C3—C4—H4	120.00
C1—N2—H2A	113.3 (18)	C5—C4—H4	120.00
C1—N2—H2B	119 (2)	N1—C5—H5	118.00
N1—C1—N2	114.6 (3)	C4—C5—H5	118.00
N1—C1—C2	119.9 (2)	C3—C6—H6A	109.00
N2—C1—C2	125.5 (2)	C3—C6—H6B	109.00
N3—C2—C1	119.4 (2)	C3—C6—H6C	109.00
N3—C2—C3	119.9 (2)	H6A—C6—H6B	109.00
C1—C2—C3	120.8 (2)	H6A—C6—H6C	109.00
C2—C3—C6	125.6 (2)	H6B—C6—H6C	109.00
			
C5—N1—C1—N2	−178.7 (2)	N2—C1—C2—N3	−2.1 (4)
C5—N1—C1—C2	2.3 (4)	N2—C1—C2—C3	177.2 (2)
C1—N1—C5—C4	0.8 (4)	N3—C2—C3—C4	−178.3 (2)
O1—N3—C2—C1	13.3 (3)	N3—C2—C3—C6	2.8 (4)
O1—N3—C2—C3	−166.0 (2)	C1—C2—C3—C4	2.4 (3)
O2—N3—C2—C1	−164.5 (2)	C1—C2—C3—C6	−176.4 (2)
O2—N3—C2—C3	16.2 (4)	C2—C3—C4—C5	0.5 (4)
N1—C1—C2—N3	176.7 (2)	C6—C3—C4—C5	179.5 (3)
N1—C1—C2—C3	−4.0 (3)	C3—C4—C5—N1	−2.3 (5)

### Hydrogen-bond geometry (Å, °)

**Table:**

*D*—H···*A*	*D*—H	H···*A*	*D*···*A*	*D*—H···*A*
N2—H2A···N1^i^	0.88 (3)	2.17 (4)	3.045 (4)	174 (3)
N2—H2B···O1	0.85 (3)	2.01 (3)	2.612 (4)	127 (2)
N3—O2···Cg1^ii^	1.203 (3)	3.2743 (3)	3.681 (12)	100.16 (17)

Symmetry codes: (i) −*x*+1, −*y*, −*z*; (ii) −*x*+2, −*y*, −*z*+1.
